# Supplementary material for: Efficient realization of quantum primitives for Shor’s algorithm using PennyLane library
Source: PLoS One. 2022 Jul 14;17(7):e0271462. doi: 10.1371/journal.pone.0271462 (PMC9282478; doi:10.1371/journal.pone.0271462)
Supplement: S1 Appendix — (PDF) [file pone.0271462.s001.pdf]

S1 Appendix. Shor's algorithm. Shor's algorithm consists of classical and quantum parts. The quantum part is used to get an efficient solution to the order-finding problem, specifically, to the task of finding the least positive integer r such that $x^r \mod N = 1$ for given positive coprime integers x and N. The scheme of the algorithm as given in Ref. [16] is shown in Fig. 9. Two theorems guarantee that the probability of success of the given algorithm is more than 0.5:

Theorem 1 [16]. Suppose $N = p_1^{\alpha_1} \dots p_m^{\alpha_m}$ is the prime factorization of an odd composite positive integer. Let x be an integer chosen uniformly at random, subject to the requirements that $1 \leq x \leq N-1$ and x is co-prime to N. Let r be the order of x modulo N. Then

$$\Pr(r \text{ is even and } x^{r/2} \neq -1 \mod N) \geq 1 - \frac{1}{2^m}. \quad (15)$$

Theorem 2 [16]. Suppose N is an L bit composite number, and y is a non-trivial solution to the equation

$$y^2 = 1 \mod N \quad (16)$$

in the range $1 \leq y \leq N$, that is, neither $y = 1 \mod N$ nor $y = N - 1 = -1 \mod N$. Then at least one of $\mathrm{GCD}(y-1, N)$ and $\mathrm{GCD}(y+1, N)$ is a non-trivial factor of N that can be computed using $O(L^3)$ operations.

After step 4 of the algorithm, Theorem 1 guarantees that the probability of the branch corresponding to the answer "no" is less than 1/2 and Theorem 2 helps to efficiently find a factor in the branch corresponding to the answer "yes". Note that all computations from the classical part of the algorithm can be efficiently performed on a classical computer.

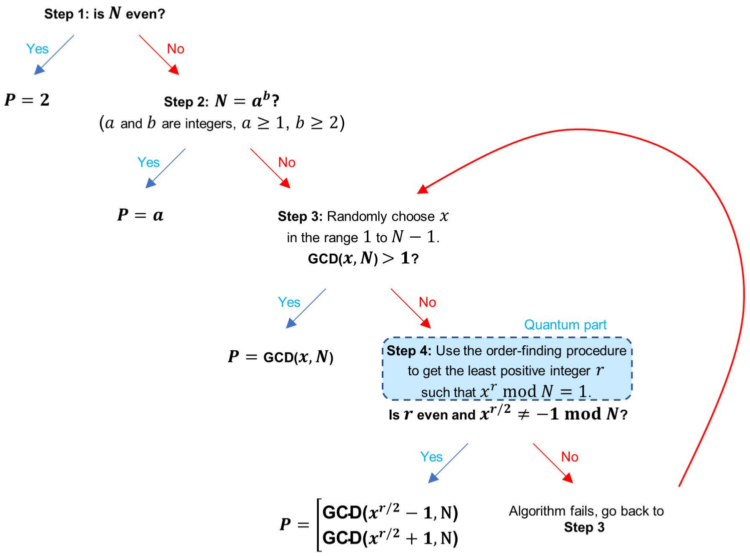

Fig 9. Shor's algorithm. The task of integer factorization is reduced to the task of order-finding that can be solved efficiently using a quantum processor.
