# Supplementary material for: Efficient realization of quantum primitives for Shor’s algorithm using PennyLane library
Source: PLoS One. 2022 Jul 14;17(7):e0271462. doi: 10.1371/journal.pone.0271462 (PMC9282478; doi:10.1371/journal.pone.0271462)
Supplement: S2 Appendix — (PDF) [file pone.0271462.s002.pdf]

S2 Appendix. Single-qubit unitary decomposition: analytic expressions.

Let us consider the decomposition of an arbitrary unitary to the sequence of two R gates:

$$\begin{aligned} U = \begin{pmatrix} u_{00} & u_{01} \\ u_{10} & u_{11} \end{pmatrix} = e^{id} R(-\pi, -c-\pi/2) R(2b+\pi, a-c-\pi/2) = \\ = e^{id} \begin{pmatrix} e^{ia}\cos b & e^{ic}\sin b \\ -e^{-ic}\sin b & e^{-ia}\cos b \end{pmatrix}. \end{aligned} \tag{17}$$

Since U is unitary, there are necessary constraints on u_{00}, u_{01}, u_{10} and d:

$$|u_{00}|^2 + |u_{01}|^2 = |u_{10}|^2 + |u_{11}|^2 = 1, \tag{18}$$

$$u_{00}u_{10}^* + u_{01}u_{11}^* = 0. \tag{19}$$

Let's prove that a, b, c and d can be uniquely defined by u_{00}, u_{01}, u_{10} and u_{11}, if these constraints on u_{00}, u_{01}, u_{10} and u_{11} are satisfied.

From the constraint (18) it follows that there exist $b, b' \in [0, \pi/2]$ s.t.

$$\begin{aligned} |u_{00}| = \cos b, \quad |u_{01}| = \sin b, \\ |u_{10}| = \sin b', \quad |u_{11}| = \cos b'. \end{aligned} \tag{20}$$

Let's assume that $b, b' \in (0, \pi/2)$, since for values 0 and $\pi/2$ decompositions in the form (17) exist. From the constraint (19) it follows that

$$\begin{aligned} & u_{00}u_{10}^* = -u_{01}u_{11}^* \Rightarrow |u_{00}u_{10}^*| = |u_{01}u_{11}^*| \Rightarrow \\ & \Rightarrow |u_{00}||u_{10}| - |u_{01}||u_{11}| = \cos b \sin b' - \sin b \cos b' = 0 \Rightarrow \\ & \Rightarrow \sin b' - b = 0 \Rightarrow \\ & \Rightarrow b = b', \text{ since } b, b' \in (0, \pi/2). \end{aligned} \tag{21}$$

Thus, for any U, there exist $b, \theta_{00}, \theta_{01}, \theta_{10}, \theta_{11}$, s.t.

$$U = \begin{pmatrix} e^{i\theta_{00}}\cos b & e^{i\theta_{01}}\sin b \\ -e^{i\theta_{10}}\sin b & e^{i\theta_{11}}\cos b \end{pmatrix}, \tag{22}$$

and analytic expression for b is

$$b = \arccos |u_{00}|. \tag{23}$$

To find analytic expressions for a, c and d, we should first find angles $\theta_{00}, \theta_{01}, \theta_{10}, \theta_{11}$ and then express a, c and d using these angles.

Note that if we divide every element of U by its absolute value, then dependency on b disappears (remember that we assumed $b \in (0, \pi/2)$, so both $\cos b$ and $\sin b$ are positive):

$$\begin{pmatrix} u_{00}/|u_{00}| & u_{01}/|u_{01}| \\ u_{10}/|u_{10}| & u_{11}/|u_{11}| \end{pmatrix} = \begin{pmatrix} e^{i\theta_{00}} & e^{i\theta_{01}} \\ -e^{i\theta_{10}} & e^{i\theta_{11}} \end{pmatrix} = \begin{pmatrix} e^{i\theta_{00}} & e^{i\theta_{01}} \\ e^{i(\theta_{10}-\pi)} & e^{i\theta_{11}} \end{pmatrix}. \tag{24}$$

Every element in this matrix has the form e^{θ}, so these angles can be expressed as

$$\begin{aligned} \theta_{00} &= \varphi_{00} + 2\pi n_{00}, & \theta_{01} &= \varphi_{01} + 2\pi n_{01}, \\ \theta_{10} &= \varphi_{10} + \pi + 2\pi n_{10}, & \theta_{11} &= \varphi_{11} + 2\pi n_{11}, \end{aligned} \tag{25}$$

where $\varphi_{ij} = \mathrm{Arg}(u_{ij})$. Referring back to the constraint (19), we get

$$\theta_{00} - \theta_{10} = \theta_{01} - \theta_{11}. \tag{26}$$

Now, if we define

$$\begin{aligned} d &= \frac{\theta_{00} + \theta_{11}}{2} = \frac{\theta_{01} + \theta_{10}}{2}, \\ a &= \frac{\theta_{00} - \theta_{11}}{2}, \\ c &= \frac{\theta_{01} - \theta_{10}}{2}, \end{aligned} \tag{27}$$

then we get the desired form (17)

We see that due to periodicity, there are many candidates for the solution.

$$\begin{aligned} d &= \frac{\theta_{00} + \theta_{11}}{2} = \frac{1}{2}(\varphi_{00} + \varphi_{11}) + \pi(n_{00} + n_{11}), \\ a &= \frac{\theta_{00} - \theta_{11}}{2} = \frac{1}{2}(\varphi_{00} - \varphi_{11}) + \pi(n_{00} - n_{11}), \\ c &= \frac{\theta_{01} - \theta_{10}}{2} = \frac{1}{2}(\varphi_{01} - \varphi_{10}) - \pi/2 + \pi(n_{01} - n_{10}). \end{aligned} \tag{28}$$

But we don't need to find all of them - just one will suffice. However, simply stating $n_{00} = n_{01} = n_{10} = n_{11} = 0$ might not work, because additionally, we have to check that the $d = \frac{\theta_{00}+\theta_{11}}{2}$ defined with elements u_{00} and u_{11} coincides with the $d = \frac{\theta_{01}+\theta_{10}}{2}$ defined with elements u_{01} and u_{10}, in accordance with the constraint (19). This might not be the case for some combinations of n_{00}, n_{01}, n_{10} and n_{11}. Since this is the only constraint on 4 variables, we can freely define three of them (for instance, $n_{00} = n_{10} = n_{11} = 0$), and the last variable will be defined from the constraint:

$$n_{01} = \frac{1}{2\pi}(\varphi_{00} - \varphi_{01} - \varphi_{10} + \varphi_{11}) - \frac{1}{2}. \tag{29}$$

To sum up, analytic expressions for a, b, c and d from (17) have the form

$$\begin{aligned} a &= \frac{1}{2}(\varphi_{00} - \varphi_{11}), \\ b &= \arccos |u_{00}|, \\ c &= \frac{1}{2}(\varphi_{00} - 2\varphi_{10} + \varphi_{11}) - \pi, \\ d &= \frac{1}{2}(\varphi_{00} + \varphi_{11}). \end{aligned} \tag{30}$$
