# Supplementary material for: Efficient realization of quantum primitives for Shor’s algorithm using PennyLane library
Source: PLoS One. 2022 Jul 14;17(7):e0271462. doi: 10.1371/journal.pone.0271462 (PMC9282478; doi:10.1371/journal.pone.0271462)
Supplement: Attachment — Submitted filename: Rebuttal Letter.pdf [file pone.0271462.s003.pdf]

Dear Editor,

We appreciate your efforts in arranging the peer review of our manuscript. We are also grateful to the Reviewers for useful suggestions and insightful comments that could help us to improve our work. We have prepared a revised version of our manuscript; we have highlighted all the changes.

Please see point-by-point answers to Reviewers below.

Reviewer #1

Question/Comment: The first requirement that the journal demands for a paper to be published is that the research must be original. This is not fulfilled since the package here presented is a direct implementation of the gates and operations described in Ref. [13], which is a 26 years old paper. No further improvement has been added, and even the figures are really similar. On the software leg, a significant contribution on computational techniques could support the publication, but this work only constructs a package on a pre-existent library and wraps the computational flow so that final results are directly obtained.

Answer: First of all, we would like to thank the Reviewer for careful reading of our manuscript and overall assessment of our work. We made some improvements, in particular, we used a simplified technique for decomposition of algorithms into a set of gates which are native for trapped-ion quantum processor and realized this technique using PennyLane library. The decomposition is used to analyze resources required for an execution of Shor's algorithm on the level of native operations of trapped-ion quantum computer in Section 5. Our original contribution is the derivation of coefficients needed for implementation of the decomposition of single-qubit operations into native rotation operations as well as proof of universality of this decomposition. These results were not given in the original work [20] containing decomposition protocol. Overall, our study shows that there is a gap between known academic results in the field of quantum information theory and implementation of quantum algorithms on available quantum platforms. And we hope that combination of realization and study of aspects of the implementation represents interesting contribution to scientific community.

Question/Comment: English and writing is also good, only a couple of informal sentences should be modified

Answer: We would be happy to edit these sentences, but we would like to know what sentences in particular did the Reviewer mention.

Question/Comment: I encourage the authors to get in contact with PennyLane and upload this package to their platform.

Answer: We’ve been in contact with PennyLane’s team, and received a recommendation to “open a PR with the contribution … It will then follow the same process that any internal contribution would, and if as you say the benefit is evident and the necessary changes are manageable, then I see no reason why this shouldn't end up in a merge”. We are going to follow this recommendation as soon as possible.

Reviewer #2

Question/Comment: The main issue is that the article fails to efficiently justify the relevance of its application. Because surely Shor’s algorithm can be implemented using other software platforms. So which is the major contribution and advantage of using the package in PennyLane? What makes the authors’ work appealing and why is it innovative, original and significant? I cannot find a direct and clear answer to these questions. Maybe it could help to specify and discuss the innovative reasons that lead the authors to create such templates

Answer: We hope that Section 5 that we have added recently will be helpful to address concerns of the Reviewer. In this section, we used a simplified technique for decomposition of algorithms into a set of gates which are native for trapped-ion quantum processor and realized this technique using PennyLane library. The decomposition is used to analyze resources required for an execution of Shor's algorithm on the level of native operations of trapped-ion quantum computer. Our original contribution is the derivation of coefficients needed for implementation of the decomposition of single-qubit operations into native rotation operations as well as proof of universality of this decomposition. These results were not given in the original work [20] containing decomposition protocol. Overall, our study shows that there is a gap between known academic results in the field of quantum information theory and implementation of quantum algorithms on available quantum platforms. And we hope that combination of realization and study of aspects of the implementation represents interesting contribution to scientific community. Additionally, the choice of PennyLane library might be supported by the ability to work with different physical realizations of quantum processors within unified framework.

Question/Comment: It would be interesting to present the limitations of the work, as well as some further applications or suggest some open questions or future problems to address. Maybe to aim or point at creating new blocks or functions, or find more algorithms to implement using PennyLane software.

Answer: An open question for further development of this work is how to define criteria for choice of algorithms for realization. Shor's algorithm has many different protocols of realizations with various advantages and disadvantages, and the protocol of the realization in our work was chosen for two reasons: efficiency of realization in terms of gate counts and simplicity of exposition. Realization of transpilation techniques could be more sophisticated as well, and it remains unclear which particular algorithms will be of greater interest in the future. We added this comment in the manuscript.

Question/Comment: Given that an example is provided [Sec.4], it could be more developed. The obtention of the final result should be more clearly explained, since this data analysis would definitely provide evidence for the stated conclusions. The results concluded from the graph of probabilities [Fig. 8] are not commented, so it would be useful to include a discussion of the final results.

Answer: Following the advice of the Reviewer, we added more elaborate explanation of the final result in the manuscript.

Question/Comment: [Table 2] shows a list of functions for auxiliary computations, but neither of these classical functions is used as an auxiliary one in the templates

presented in the document, and they are not even mentioned. Maybe it would be nice to state where and how these functions are being used and for what purpose.

Answer: Classical function modular_multiplicative_inverse is crucial for building the decomposition and it is used as the auxiliary function for MODULAR_EXPONENTIATION. The role of this classical function is to find parameters for the lower-level decomposition Ctrl_MULT_MOD_inv. Important aspect of modular_multiplicative_inverse is the efficiency of the realization that relies on the efficiency of two other classical functions gcd and diophantine_equation. We added this comment in the manuscript.

Question/Comment: Specially, a really effective point is the pairing of the schematic circuit with its analogue coding implementation, since they complement each other. This connection allows a correct comprehension both of the programming and the functionality of the circuit, so that the redundancy acts as a strength mechanism for reinforcing the reader's understanding. Also, the use of these resources enables the authors to support their claims, since they provide evidence for the authors' main findings and developments. Therefore, I recommend this manuscript to be published in PLOS ONE.

Answer: We are grateful to the Reviewer for the appreciation of our work!

With kind regards,
The authors.
